# Idiopathic nephrotic syndrome in South African children

**DOI:** 10.4314/ahs.v17i4.22

**Published:** 2017-12

**Authors:** Yassir Mahgoub Bakhiet, Abdullahi Mudi, Tholang Khumalo, Glenda Moonsamy, Cecil Levy

**Affiliations:** 1 Division of Paediatric Nephrology, Department of Paediatrics and Child Health, Charlotte Maxeke Johannesburg Academic Hospital, Johannesburg, South Africa; 2 Faculty of Health Sciences, University of the Witwatersrand, Johannesburg, South Africa; 3 Department of Paediatrics, Faculty of Clinical Sciences, College of Health Sciences, Bayero University, Kano, Nigeria

**Keywords:** Idiopathic, nephrotic syndrome, children

## Abstract

**Background:**

Different histo-pathological types and treatment response patterns of Idiopathic nephrotic syndrome (INS) have been associated with differences in ethnicity and geographical location.

**Objective:**

To provide an update on the steroid response and renal histo-pathological pattern in children treated for INS.

**Method:**

Medical records of children with INS treated at the Charlotte Maxeke Johannesburg Academic Hospital were reviewed.

**Results:**

Mean age was 5.3 years ± 2.8. The majority (68.1%) of the 163 children were of the black racial group. The highest rate of INS was seen in the 2–6 year age group (71.2%). The black racial group had the highest rate (42/111; 37.8%) of focal segmental glomerulosclerosis (FSGS), and the white race had the highest rate (9/14; 64.3%) of minimal change disease (MCD). Ninety four (57.7%) patients were steroid sensitive (SSNS) while 69 patients (42.3%) were steroid resistant (SRNS). Minimal change disease was the most common histo-pathological type seen in SSNS (60%), while FSGS was the most common observed in patients who had SRNS (65.2%).

**Conclusion:**

There appears to be a higher rate of FSGS in all the racial groups, and also a higher rate of MCD in the black race group, when compared to previous reports.

## Introduction

The spectrum of idiopathic nephrotic syndrome (INS) in African countries seems to be different from other parts of the world suggesting that an interaction of genetic and environmental factors plays an important role in the pathogenesis of INS^1^,^2^. Worldwide, a significant number of cases of INS are usually steroid sensitive, while some cases will be diagnosed as having steroid unresponsive disease^3^–^5^.

The histo-pathological pattern of presentation, its changing pattern and advances in the treatment of childhood INS have been reported widely^6^–^12^. Childhood INS is most commonly caused by one of several idiopathic diseases: Minimal-change disease (MCD), focal segmental glomerulosclerosis (FSGS) and, less commonly, idiopathic mesangial proliferative glomerulonephritis (MesPGN)^3^,^4^,^13^. These various histo-pathological features have been strongly associated with differences in response to corticosteroid therapy, subsequent clinical course and prognosis^4^,^13^. The general impression is that the histo-pathological pattern of INS has been changing despite a stable incidence of the disease in the last 30 years. This is as noted by the increasing reported incidence of FSGS^6^–^14^–^16^. Idiopathic nephrotic syndrome remains an important cause of childhood glomerulopathy. A better understanding of the presentation, the steroid response pattern and the histo-pathological pattern of childhood INS is important in determining future guidelines for effective management of the disease.

In this study, we aimed to describe the steroid response and renal histo-pathological pattern in a cohort of children treated for INS in the Division of Paediatric Nephrology, Charlotte Maxeke Johannesburg Academic Hospital, Johannesburg, South Africa.

## Methods

This retrospective study reviewed children (2–16 years) with INS treated by the Division of Paediatric Nephrology at Charlotte Maxeke Johannesburg Academic Hospital between January 2004 and December 2013. Only children who had at least 6 months of follow up after onset of the disease were included in the study. Children with evidence of chronic renal impairment prior to the diagnosis of INS (estimated glomerular filtration rate (eGFR) ≤60 mL/min/1.73 m^2^) were excluded from the study. Demographic and clinical information were obtained from the patients' records.

Additional information on renal biopsies were obtained from the records. The renal biopsies were carried out under ultrasound guidance and the indications for renal biopsy were steroid resistance, frequent relapse, steroid dependence and an atypical presentation such as the presence of hypertension, haematuria on presentation, or age greater than, or equal to, 10 years. The renal tissues were subject to light microscopy, immunofluorescence and electron microscopy.

## Data analysis

All data were collated, checked and analysed using a computer based statistical package STATA version 13.1. Continuous parameters were reported as mean and standard deviation while categorical variables were presented as percentages and bar charts. Associations between groups were determined using chi-square testing and bilateral Fisher's exact tests. A p value < 0.05 was regarded as statistically significant.

## Ethics and permission

The study was approved by the University of the Witwatersrand, Human Research Ethics Committee (Protocol M150234) and was conducted in conformance with the Helsinki Declaration, Good Clinical Practice and within the laws and regulations of South Africa.

## Definition of terms

**1. Steroid response pattern**^17^:

- *Steroid sensitive nephrotic syndrome* (SSNS) (initial responder): Attainment of remission within initial 4 weeks of corticosteroid therapy.
- *Steroid dependent nephrotic syndrome* (SDNS): Two consecutive relapses during corticosteroid therapy, or within 14 days of ceasing therapy.
- *Steroid resistant nephrotic syndrome* (SRNS) (initial non-responder/steroid resistance): Failure to achieve complete remission after 8 weeks of corticosteroid therapy.

**2. Remission**^17^: Urine protein/creatinine ratio (uPCR) < 200 mg/g (< 0.02 g/mmol) or <1+ of protein on urine dipstick for 3 consecutive days.

**3. Relapse**^17^: Urine PCR ≥ 2000 mg/g (≥ 0.2 g/mmol) or ≥ 3+ protein on urine dipstick for 3 consecutive days:

- *Infrequently relapsing nephrotic syndrome* (IFRNS): One relapse within 6 months of initial response, or one to three relapses in any 12 month period.
- *Frequently relapsing nephrotic syndrome* (FRNS): Two or more relapses within 6 months of initial response, or four or more relapses in any 12 month period.

## Results

A total of 163 children who met the study criteria were recruited. There were 97 (59.5%) males and 66 (40.5%) females with a male to female ratio of 1.5:1. The mean age of the children at the time of presentation was 5.3 years ± 2.8. The mean follow-up period was 60 months ± 36.9.

At presentation, the majority of the patients were in the 2–6 year age group (71.2%) (Table 1). The majority of the patients in the cohort were of the black race (68.1%) and the black race made up the highest number in all the age groups (Table 1). Common features at presentation include hypercholesterolaemia (122; 74.8%), hypertension (77; 47.2%), haematuria (32; 19.6%) and infections (respiratory tract infection 35, peritonitis 21 and UTI 3).

**Table 1:** The age group distribution according to the racial groups

	Racial group (%)				
Age group (year)	Black	Asian	Mixed Race	White	Total
**2 – 6**	71 (61.2)	20 (17.2)	14 (12.1)	11 (9.5)	116 (100)
**7 – 10**	30 (83.3)	1 (2.8)	2 (5.6)	3 (8.3)	36 (100)
**11 – 16**	10 (90.9)	1 (9.1)	0 (0.0)	0 (0.0)	11 (100)

One hundred and thirty-two (80.9%) of the patients underwent a renal biopsy. The indications for renal biopsy can be seen in Figure 1. The most common histopathological lesion observed was MCD (52.3%) (Table 2). The mean age at presentation of MCD and FSGS was similar, 64.5 months ± 28.3 and 67.0 months ± 39.8 respectively. The mean age at presentation for MesPGN was higher (94.0 months ± 28.6) when compared to the others. The black race had a similar rate of MCD (38.8%) and FSGS (37.8%) while the white race had a higher rate of MCD (64.3%) when compared to FSGS (14.3%) (Table 2).

**Table 2:** The different histopathological lesions

	No Biopsy	MCD	FSGS	MesPGN	Total
**Age group (year)**					
**2 – 6**	26 (22.4)	50 (43.1)	37 (31.9)	3 ( 2.6)	116 (100)
**7 – 10**	4 (11.1)	17 (47.2)	13 (36.1)	2 (5.6)	36 (100)
**11 – 16**	1 (9.1)	2 (18.2)	7 (63.6)	1 (9.1)	11 (100)
					**p=0.116**
**Racial group**					
**Black**	21 (18.9)	43 (38.8)	42 (37.8)	5 (4.5)	111 (100)
**Asian**	6 (27.3)	9 (40.9)	7 (31.8)	0 (0.0)	22 (100)
**Mixed**	2 (12.5)	8 (50.0)	6 (37.5)	0 (0.0)	16 (100)
**White**	2 (14.3)	9 (64.3)	2 (14.3)	1 (7.1)	14 (100)
					**p=0.604**
**Clinical type**					
**SSNS**	20 (30.8)	39 (60)	6 (9.2)	0 (0.0)	65 (100)
**SDNS**	5 (17.2)	18 (62.1)	6 (20.7)	0 (0.0)	29 (100)
**SRNS**	6 (8.7)	12 (17.4)	45 (65.2)	6 (8.7)	69 (100)
					**p<0.001**

All of the patients received prednisolone 2 mg/kg as a daily dose for at least 4 weeks. The steroid response pattern was as follows; 94/163 (57.7%) patients had SSNS, and SRNS was present in 69/163 patients (42.3%). Of the patients who were initially steroid sensitive, 55/94 (58.5%) turned out to be infrequent relapsers, 10/94 (10.6%) became frequent relapsers and 29/94 (30.9%) became steroid dependent.

Minimal change disease was the most common histopathological lesion (60%) seen in SSNS, while FSGS was the most common lesion (65.2%) observed in patients who had SRNS. Steroid sensitive NS was seen more in the Asian (59.1%) and white groups (50%) while the mixed race and black racial groups had a higher incidence of SRNS (50% and 46% respectively) (Table 3).

**Table 3:** The relationship between the racial groups and the steroid response patterns

	Steroid response pattern (%)			
Racial group	SSNS	SDNS	SRNS	Total
**Black**	39 (35.1)	21 (18.9)	51 (46.0)	111 (100)
**Asian**	13 (59.1)	3 (13.6)	6 (27.3)	22 (100)
**Mixed**	6 (37.5)	2 (12.5)	8 (50.0)	16 (100)
**White**	7 (50.0)	3 (21.4)	4 (28.6)	14 (100)
**Total**	65 (39.9)	29 (17.8)	69 (42.3)	163 (100)
				**p=0.434**

Steroid sparing drugs (SSDs) such as alkylating agent (Cyclophosphamide), calcineurin inhibitors (Cyclosporin, Tacrolimus) and antiproliferative agents (Mycophenolate Mofetil) were used in patients who were steroid dependent and steroid resistant. Nine of the SRNS patients were able to achieve remission with the use of SSDs. Seven of these remained in remission afterwards on SSDs and corticosteroid treatment, while two developed a frequently relapsing picture while on SSDs and corticosteroid treatment (Table 4).

**Table 4:** The outcome of INS at a mean follow up period of 60 months

	Sustained remission without any treatment	Remission on steroid treatment alone	Remission on SSDs and steroid treatment	FRNS	Resistant to all treatment	Total
**Steroid** **response**						
**SSNS**	53 (81.5)	1 (1.5)	0 (0.0)	10 (15.5)	1 (1.5)	65 (100.0)
**SDNS**	0 (0.0)	27 (93.1)	2 (6.9)	0 (0.0)	0 (0.0)	29 (100.0)
**SRNS**	0 (0.0)	0 (0.0)	7 (10.1)	2 (2.9)	60 (87.0)	69 (100.0)
						**p<0.001**

**Racial group**						
**Black**	34 (30.7)	22 (19.8)	5 (4.5)	5 (4.5)	45 (40.5)	111 (100.0)
**Asian**	11 (50.0)	2 (9.1)	1 (4.6)	3 (13.6)	5 (22.7)	22 (100.0)
**Mixed**	3 (18.7)	1 (6.3)	2 (12.5)	2 (12.5)	8 (50.0)	16 (100.0)
**White**	5 (35.7)	3 (21.4)	1 (7.2)	2 (14.3)	3 (21.4)	14 (100.0)
						**p=0.135**

The highest rate of resistance to all treatment after a mean follow up of 60 months was seen among children of the mixed race and black racial groups (50.0% and 40.5% respectively) (Table 4).

## Discussion

This study found a mean age at presentation that is similar to previous studies^1^,^6^,^7^,^9^,^12^,^18^,^19^, but lower than that previously reported by Bhimma et al in Durban, South Africa^1^. Although we expected our study cohort to have a similar age of presentation to that of the Durban study, the Durban study had a higher percentage of Asian patients in their cohort than we had in ours, and this may explain why their mean age of presentation was closer to that observed in studies from India and Pakistan than to that of our study^18^,^20^.

In our study, black children made up the predominant race group (68.1%) and this is similar to a previous study from Pretoria, South Africa that reported a cohort made up predominantly of black patients (77.7%)^6^. The study from Pretoria had only two racial groups in the study, white and black, and this may explain the greater percentage of black patients reported in their findings as compared to our study. On the contrary, the study from Durban reported the Indian (Asian) race to be the predominant group (52.5%), with the black race representing only 43.3% of the patients^1^.

A high rate of hypertension (47.2%) was recorded as one of the common presenting features of our patients. This is not in keeping with the majority of studies which have specifically looked at children with INS (15–31%)^9^–^10^–^20^–^23^, and may be related to the higher rate of FSGS (43.2%) documented in our study. Furthermore, a large number of our patients (68.1%) were of the black racial group when compared to the other studies, and the risk of hypertension has been associated with the black race in the past^24^–^26^.

Minimal change disease (MCD) accounted for the most common histo-pathologic variant observed in our cohort (52.3%). This is similar to the report from Pakistan where MCD accounted for 51.2%^20^. Other studies, including previous South African studies, have reported a much lower rate of MCD (<36%)^1^,^6^,^12^,^18^,^19^. It is possible that these apparent differences in the rate of MCD in children may be due to variations in the indications for biopsy in the different studies (where the most common indication was steroid resistance), rather than actual differences in the rate of MCD in the various population groups studied.

Focal segmental glomerulosclerosis (FSGS) was found to have a rate of 43.2% in our study which is higher than the rates reported in previous South African studies namely 28.5% in the Durban study, 25% in the Pretoria study and 31.3% in a study from Johannesburg^1^,^6^,^27^. One possible reason for this finding may be the decline of infection related nephrotic syndrome (NS). Up until now this was one of the most common causes of NS in children and presented as other histo-pathological sub-types such as Membranous Nephropathy (MN) and Membranoproliferative glomerulonephritis (MPGN)^1^. Other possible reasons include the theory that suggests the transition of MCD to FSGS in selected cases, although this is yet to be proven, and the very real possibility of misdiagnosing FSGS as MCD due to technical challenges or sampling error^28^,^29^. As has been also noted in previous studies, in our cohort, the age of presentation of FSGS was most common in the age group of 2–6 years^1^,^12^.

Mesangial proliferative glomerulonephritis (MesPGN) was the least common histo-pathologic variant observed in our study (4.5%). Even though higher rates have been reported from India (11.4%), Turkey (17%) and the US (25%), our findings are in keeping with rates reported in previous South African studies^1^,^6^,^10^,^12^,^18^.

Ethnicity has been implicated in playing a role in the histopathological types of INS. Studies with diverse patient cohorts have reported higher rates of FSGS in the black race group^1^,^6^,^10^. Similarly, in our group FSGS made up a larger proportion of cases in the black race group when compared to the other race groups.

Despite the inhomogeneous nature of our cohort, we observed an initial response rate to steroid therapy of 57.7% and a steroid resistance rate of 42.3%. This is almost similar to the steroid response pattern reported from Pakistan, even though they had a much more homogeneous cohort^20^. Studies from New Zealand and Iran reported much higher steroid response rates of 80.4% and 75.2% respectively^22^,^23^. A similar rate of frequent relapse (10.6%) and steroid dependence (17.8%) was observed in our steroid sensitive group of patients to that which was reported in the study from New Zealand^23^.

Although we observed a high rate of steroid resistance among the black racial group in our study, this group also had a high rate of steroid sensitivity (35.1%). This is contrary to reports from previous South African studies and the traditional belief that most black children are steroid resistant^1^,^6^,^27^. We also observed that the mixed racial group exhibited a similar steroid response pattern to the black racial group.

A small fraction of the MCD group was steroid resistant (17.4%), and likewise a small fraction of the FSGS group was steroid sensitive (21%). These findings are in keeping with findings reported elsewhere^12^,^18^,^20^,^22^,^23^.

## Strength and limitations

This study provides an update on children with INS and also highlights the higher rate of FSGS in all racial groups when compared to previous studies. The main limitations of our study are that it is retrospective and that we could not compare the response rate to other agents used in SRNS.

## Conclusion

We found higher rates of MCD, and also of favourable steroid response patterns, in our black race group than have been previously reported from South Africa. Of concern is our finding of a higher rate of FSGS in all the racial groups when compared to previous studies and we are not sure if this represents a new trend that FSGS is increasing in our population. If our finding of a higher rate of FSGS when compared to previous South African studies is real, this may mean that in the future we might begin to see an increase in the rate of steroid resistance among South African children with INS.

We suggest a prospective, multicentre study on childhood INS in Africa for better understanding of the disease in order to tailor treatment regimens unique to our patients.

## Role of authors

Bakhiet: Conception, data collection, data analysis and interpretation, drafting, final approval for submission

Mudi: Conception, data analysis and interpretation, drafting, critical review, final approval for submission

Khumalo: Conception, data analysis and interpretation, drafting, critical review, final approval for submission

Moonsamy: Conception, data analysis and interpretation, drafting, critical review, final approval for submission

Levy: Conception, data analysis and interpretation, drafting, critical review, final approval for submission
